# Role of *CCL3L1-CCR5* Genotypes in the Epidemic Spread of HIV-1 and Evaluation of Vaccine Efficacy

**DOI:** 10.1371/journal.pone.0003671

**Published:** 2008-11-07

**Authors:** Hemant Kulkarni, Vincent C. Marconi, Brian K. Agan, Carole McArthur, George Crawford, Robert A. Clark, Matthew J. Dolan, Sunil K. Ahuja

**Affiliations:** 1 Veterans Administration Research Center for AIDS and HIV-1 Infection, South Texas Veterans Health Care System, San Antonio, Texas, United States of America; 2 Infectious Disease Clinical Research Program, Uniformed Services University of the Health Sciences, Bethesda, Maryland, United States of America; 3 Infectious Disease Service, Wilford Hall United States Air Force Medical Center, Lackland Air Force Base, Texas, United States of America; 4 San Antonio Military Medical Center, Fort Sam Houston, Texas, United States of America; 5 Department of Oral Biology, School of Dentistry, University of Missouri-Kansas City, Kansas City, Missouri, United States of America; 6 Henry M. Jackson Foundation, Wilford Hall United States Air Force Medical Center, Lackland Air Force Base, Texas, United States of America; 7 Department of Medicine, Microbiology and Immunology and Biochemistry, University of Texas Health Science Center, San Antonio, Texas, United States of America; New York University School of Medicine, United States of America

## Abstract

**Background:**

Polymorphisms in CCR5, the major coreceptor for HIV, and CCL3L1, a potent CCR5 ligand and HIV-suppressive chemokine, are determinants of HIV-AIDS susceptibility. Here, we mathematically modeled the potential impact of these genetic factors on the epidemic spread of HIV, as well as on its prevention.

**Methods and Results:**

*Ro*, the basic reproductive number, is a fundamental concept in explaining the emergence and persistence of epidemics. By modeling sexual transmission among HIV+/HIV− partner pairs, we find that *Ro* estimates, and concordantly, the temporal and spatial patterns of HIV outgrowth are highly dependent on the infecting partners' *CCL3L1-CCR5* genotype. *Ro* was least and highest when the infected partner possessed protective and detrimental *CCL3L1-CCR5* genotypes, respectively. The modeling data indicate that in populations such as Pygmies with a high *CCL3L1* gene dose and protective *CCR5* genotypes, the spread of HIV might be minimal. Additionally, *Pc*, the critical vaccination proportion, an estimate of the fraction of the population that must be vaccinated successfully to eradicate an epidemic was <1 only when the infected partner had a protective *CCL3L1-CCR5* genotype. Since in practice *Pc* cannot be >1, to prevent epidemic spread, population groups defined by specific *CCL3L1-CCR5* genotypes might require repeated vaccination, or as our models suggest, a vaccine with an efficacy of >70%. Further, failure to account for *CCL3L1-CCR5*-based genetic risk might confound estimates of vaccine efficacy. For example, in a modeled trial of 500 subjects, misallocation of *CCL3L1-CCR5* genotype of only 25 (5%) subjects between placebo and vaccine arms results in a relative error of ∼12% from the true vaccine efficacy.

**Conclusions:**

*CCL3L1-CCR5* genotypes may impact on the dynamics of the HIV epidemic and, consequently, the observed heterogeneous global distribution of HIV infection. As *Ro* is lowest when the infecting partner has beneficial *CCL3L1-CCR5* genotypes, we infer that therapeutic vaccines directed towards reducing the infectivity of the host may play a role in halting epidemic spread. Further, *CCL3L1-CCR5* genotype may provide critical guidance for optimizing the design and evaluation of HIV-1 vaccine trials and prevention programs.

## Introduction

For more than 25 years, HIV-1 infection has been spreading across human populations relentlessly. An improved understanding of the factors that promote viral spread and an effective vaccine is required to halt this pandemic. Significant attention has been placed on elucidating the impact of the HIV-1 genotype on the spread of infection and on development of an HIV vaccine. Although much less is known about the impact of host factors on these events, several reasons suggest that their contribution might be large. The spread of HIV in the general population is a product of the susceptibility of uninfected persons and the communicability of HIV from the infected person [1], [2], [3]. This communicability is, in part, reflected by infectivity of the host as measured by the plasma RNA viral load (VL) [1], [2], [3], [4], [5], [6]. However, we and others have demonstrated that susceptibility and communicability are dictated, in part, by polymorphisms in genes that influence HIV-AIDS susceptibility ([7], [8], [9], [10], [11], [12], [13], [14], [15], [16], [17], [18], [19], [20], [21], [22], [23] and reviewed in [24], [25], [26], [27], [28], [29]). For this reason, we hypothesized that the inherent variability among individuals in host genes that influence HIV-AIDS susceptibility, when translated to the level of a population, might influence the epidemic spread of HIV in that population and, by extension, might contribute to the observed heterogeneous distribution of HIV among populations [3], [30], [31], [32], [33], [34], [35], [36], [37], [38]. We also posited that if evidence in support of this hypothesis were to be found, it might have implications for the possibility that failure to account for host factors that influence HIV-AIDS susceptibility may pose a challenge in designing public health measures to curb the epidemic, including evaluation of the efficacy of a vaccine. This was relevant in light of data from a recent HIV vaccine trial where vaccination was associated with an increased risk of acquiring HIV infection [39], [40], [41], [42], [43], [44].

We selected two candidate genes to test our hypotheses: those coding for CC chemokine receptor 5 (CCR5), the major HIV coreceptor [45], [46], and CC chemokine ligand 3-like 1 (CCL3L1), the most potent CCR5 ligand and HIV-suppressive chemokine [47], [48], [49], [50], [51], [52]. In previous studies, we and others found that the copy number of the *CCL3L1*-containing segmental duplications and/or genotypes of *CCR5* were determinants of inter-individual differences in several parameters: cell-mediated immunity (CMI) as assessed by delayed-type hypersensitivity (DTH) skin test reactivity in both HIV-negative and -positive individuals [53]; HIV acquisition [7], [8], [9], [20], [54], [55], [56], [57]; events established during the early stages of the infection such as the magnitude of initial CD4+ T-cell depletion and the extent of viral replication as reflected by the steady-state plasma HIV RNA VL (VL setpoint) [20], [53], [58]; rate and extent of CD4+ T cell depletion during disease course and, consequently, risk and rate of AIDS development [7], [8], [10], [11], [13], [14], [15], [20], [24], [25], [26], [27], [28], [53], [58] and recovery of CD4+ T cells during HAART [58], [59], [60], [61]. Others have also found a relationship between the copy number of *CCL3L1*-containing segmental duplications and viral load and HIV-specific CD4+ and CD8+ T cell responses [55], [62]. However, we also found that the *CCL3L1* gene dose and *CCR5* genotypes affected risk and rate of developing AIDS independent of their effects on the VL or CMI as assessed by DTH skin test reactivity [53], [63]. The latter findings indicated that only a portion of the disease-modifying effects associated with these two host factors on HIV-AIDS susceptibility can be captured by assessing the plasma VL or a surrogate marker of CMI. The nature of these unmeasured effects is currently unknown.

Factors that influence the magnitude of the events that are established during the early stages of infection have important public health relevance, as the rate of transmission is highest during this phase of the infection [3], [64]. Furthermore, CMI is a critical antiviral host response [65]. Thus, we surmised that by affecting CMI in HIV-negative and -positive individuals, viral entry, early events, and other unmeasured parameters, *CCL3L1-CCR5* genotypes might affect risk of HIV acquisition and disease progression rates for an individual patient, while at the level of populations they might modify the propagation and maintenance of the HIV pandemic and consequently, they may impact on preventive interventions. To examine this possibility, we used modeling approaches to determine whether genetic variations in *CCL3L1-CCR5* can serve as a biological basis for distinct subtrajectories of the HIV epidemic in a population, and whether accounting for the *CCL3L1-CCR5* genetic makeup of vaccine trials could provide a more precise estimate of the critical proportion of the population-based vaccination coverage required to limit the epidemic, as well as vaccine efficacy.

## Results

### Modeled impact of CCL3L1-CCR5 genetic risk groups (GRG) on the epidemic

**Figure 1:**
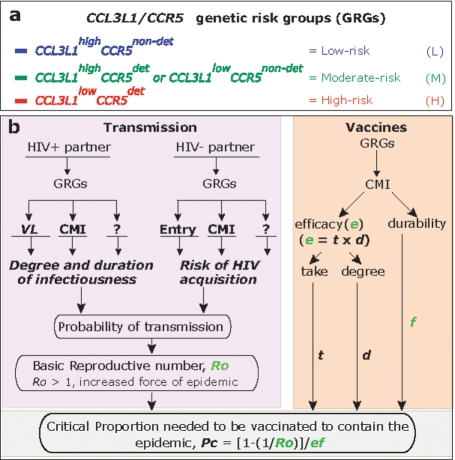
Conceptual model by which *CCL3L1-CCR5* genotypes might influence the prevention and epidemic outgrowth of HIV infection. (a) Classification system used to categorize the copy number of *CCL3L1* (low or high) and genotypes of *CCR5* (detrimental or nondetrimental) into three (low, moderate and high) risk categories. (b) Conceptual model by which the GRGs might affect epidemiological endpoints. The endpoint *Pc* has different components, and those that might be influenced by the GRGs are shown in green-colored letters, i.e., *Ro*, *e* and *f*. The model assumptions, parameters and methods are described in Supplementary Online Materials S1 (SOM), section 1 and Table S1. The conceptual model assumes a vaccine that requires the induction of CMI, in part, for protection. Here, the formula of *Pc* is from studies by Anderson [1] and Blower [2]. ?, indicates possible additional effects associated with *CCL3L1-CCR5* GRGs that are unmeasured [53], [63].

Based on possession of a low or high copy number of *CCL3L1* (*CCL3L1^low^* or *CCL3L1^high^*) and detrimental or nondetrimental *CCR5* genotypes (*CCR5^det^* and *CCR5^non-det^*) we assigned the polymorphisms in these two genes into four groups [20]. Based on their associations with a low, moderate and high risk for disease progression rates to AIDS and death in a portion of the U.S.-based DoD HIV Natural History Study cohort [20], we designated these genotypes as low, moderate and high genetic risk groups (GRG; **Figure 1a**) [53], [58].

We examined the population-level impact of *CCL3L1-CCR5* GRG status within the rubric of an epidemiologic parameter, designated as *Pc*, as well as other relevant epidemiologic parameters. The description of these parameters and their derivations are described in **Supplementary online material S1 (SOM) section 1**. The critical vaccination proportion or *Pc* is an important estimate of the fraction of the population that must be vaccinated successfully to eradicate an epidemic. The mathematical components of *Pc* are shown in **Figure 1b** along with the possible points at which GRG status might affect this estimate (**Figure 1b** and **Table S1** online). The model in Figure 1b shows that the GRGs might influence the estimate of *Pc* primarily by influencing infectiousness and susceptibility, and possibly by also affecting responses to vaccines that rely on the generation of CMI. One important component of *Pc* is the basic reproductive number (*Ro*) (**Figure 1b**). Effective human-to-human transmission of an infectious agent requires that *Ro* should exceed one, where *Ro* is the average number of secondary infections arising from one infected individual in a completely susceptible population [66]. Thus, the epidemic threshold is *Ro* = 1, above which the disease spreads and below which it eventually dies out. Given that variations in *CCL3L1-CCR5* are associated with altered HIV susceptibility and/or communicability, we first used highly conservative assumptions to model the effects of the *CCL3L1-CCR5* GRGs on *Ro*.

**Figure 2:**
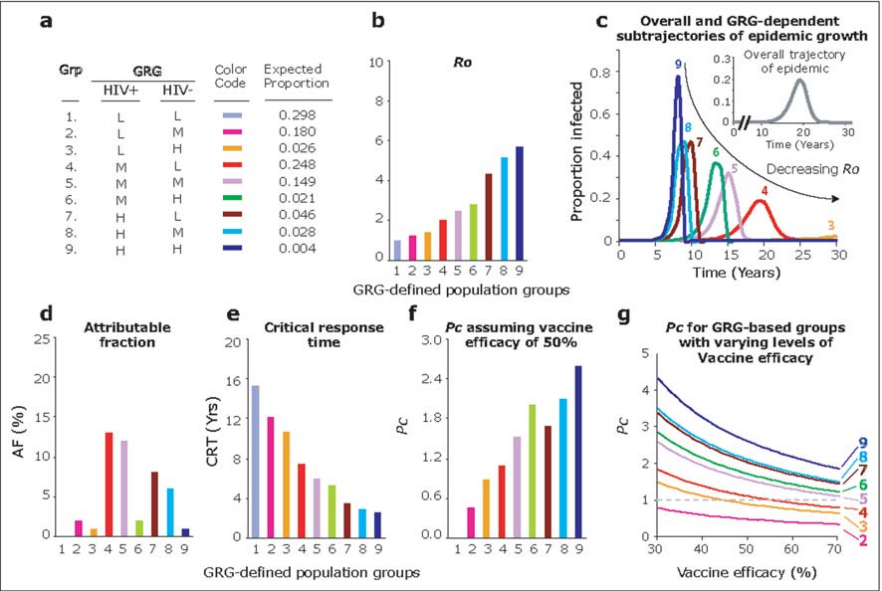
Modeling studies assessing the influence of *CCL3L1-CCR5* genotypes on epidemiological parameters relevant to the outgrowth and prevention of HIV-1. (a) Nine population groups based on the *CCL3L1-CCR5* GRG status of sexual partners. The estimated proportions (prevalence) of the GRGs in the general population are based on data from the HIV-positive WHMC cohort [20]. L, M, and H denote low, moderate, and high GRG status, respectively. The color codes shown are used to illustrate the nine population groups (Grp) in panels b to g. (b) Estimates of *Ro* for the nine color-coded GRG-defined population groups. (c) Simulated epidemic growth in GRG-defined population groups. Methods are in SOM, section 1.9 online. (d to f) Attributable fractions (AF, panel d), critical response time (CRT, panel e) and *Pc* (f) in the nine population groups. The calculations for *Pc* (f) assume a vaccine efficacy of 50%. (g) Influence of varying vaccine efficacy estimates on *Pc* in the nine GRG-defined population. groups shown in panel a. *Pc* values greater than unity (dashed horizontal line) indicate the point at which repeated mass vaccination might be necessary. *Pc* for population group #1 was zero. Additional data relevant to these studies are shown in Table S2 online.

We modeled sexual transmission, and based on the GRG status of the infected and uninfected partner pair, the overall population can be divided into nine partner-pair population groups (**Figure 2a**). We determined the distribution of the GRG-defined partner pairs from the prevalence (parenthesis) of the low (50%), moderate (42%) and high (8%) GRG status in a large US-based cohort of mixed ethnicity [20]. Assuming random mixing among individuals in the general population, the GRG-defined partner pairs in which one member has a low and moderate GRG constituted nearly 88% of the population, i.e., group numbers 1, 2, 4 and 5 (**Figure 2a**). In all but one (group #1) of these nine groups, the *Ro* was greater than unity (**Figure 2b**). In partner-pair groups 1 to 9, *Ro* increased in a step-wise manner, and this increase was mostly dependent on the GRG status of the infected partner (**Figure 2b**). HIV+/HIV− partner pairs defined by a low-low, moderate-moderate, and high-high GRG status — that is, partner-pair population groups 1, 5 and 9 — were associated with the least, intermediate and highest *Ro*, respectively (**Figure 2a** and **Figure 2b**).

Since *Ro* is a fundamental concept in explaining the emergence and persistence of epidemics, we next determined whether these GRG-dependent differences in *Ro* might also translate into differences in the rate of epidemic growth (the number of new infected cases per unit of time) in partner-pair population groups categorized based on their *CCL3L1-CCR5* GRG status (**Figure 2c**). In a model that assumes a closed population, the simulated overall trajectory of the epidemic growth took approximately ten years to emerge and, without an influx of a susceptible pool of individuals, predictably, the simulated epidemic eventually died out (**Figure 2c**, inset). However, the simulated trajectories for each of the nine GRG-defined population groups were strikingly different in three ways: the time point at which an increase in epidemic growth became evident; the extent of epidemic growth, i.e., the proportion of subjects infected; and the duration for which the epidemic persisted (**Figure 2c**). These differences in the trajectories in large part tracked the *Ro* estimates and were predictably highly dependent on the infecting partner's GRG status (**Figure 2c**). Remarkably, the epidemic growth was negligible when the infected partner possessed a low GRG (groups 1 to 3; **Figure 2c**). Conversely, the epidemic outgrowth was maximal when the HIV-infected partner pair possessed a high GRG (groups 7 to 9; **Figure 2c**).

The nine GRG-defined population groups together contributed approximately 45% to the overall epidemic growth (as estimated by attributable fraction (AF); **Figure 2d**). However, the relative contribution of each of the nine GRG-defined population groups to the overall epidemic varied significantly (0 to 13%). Consistent with findings shown in Figure 2c, the highest AFs, and consequently the greatest contribution to the overall epidemic, were due to infected subjects possessing the moderate or high GRGs (groups 4 to 9; **Figure 2d**), i.e., those who are more likely to be infectious because of higher VLs or have greater susceptibility to acquiring virus [20], [53]. This implies that a reduction in the population-level viral load – for example, by a vaccine or HAART – will have the greatest benefit in abating the spread of the epidemic when applied to these *CCL3L1-CCR5* genetically-defined subjects which agrees with the growing view that a vaccine that can modify disease course by lowering the viral load setpoint holds promise in curbing epidemic growth of HIV [1], [2], [41], [67], [68], [69]. The impact of this strategy could be large, since these subjects make up nearly 50% of the infected population (**Figure 2a**). Additionally, the critical response time (CRT), defined as the time interval within which the number of epidemic cases remains stationary (so that interventions implemented within CRT may be the most effective or the least costly), varied across the GRGs from 2.59 to 15.3 years (**Figure 2e**). Thus, these modeling data suggest that the time available to implement control measures against spread of the infection may also be highly dependent on the GRGs.

Finally, using highly conservative assumptions, we considered the effect of the *CCL3L1-CCR5* GRGs on the estimate of *Pc*. In sensitivity analyses, we found that *Pc* was much more sensitive to changes in the values of *Ro* than those of vaccine take (*t*) or durability (*d*) (**Figure 1b**) (**SOM**, **section 1.5** online). This implies that, regardless of the effects of variations in *CCL3L1-CCR5* on the eventual immune responses induced by a vaccine, the bulk of the effects of these genetic factors on *Pc* will be due to their effects on *Ro*. Consistent with this, in a mathematical model in which the values of vaccine take and durability were held constant, and assuming a vaccine that has an efficacy of 50%, the *Pc* estimates increased with increasing values of *Ro* (**Figure 2f**). *Pc* was less than unity in the population groups in which the infected partner possessed a low GRG. Remarkably, *Pc* was zero when the both the infected and uninfected partner pair possessed a low GRG, suggesting that from a purely theoretical perspective this population group, which constitutes ∼30% of the overall population (**Figure 2a**), may not require vaccination. By contrast, *Pc* was greater than unity in the six population groups in which the infected partner possessed a moderate or high GRG.

Since in practice *Pc* cannot be greater than unity, this implies that to contain the epidemic in population groups where *Pc*>1 might require repeated immunizations, or vaccination with a more efficacious vaccine (**Figure 2g**). For example, our models indicate that a vaccine with an efficacy of more than 70% would be required to confer protection by mass vaccination in population groups 5 to 9 (**Figure 2g**).

### Impact of GRGs on assessment of HIV vaccine efficacy

**Figure 3:**
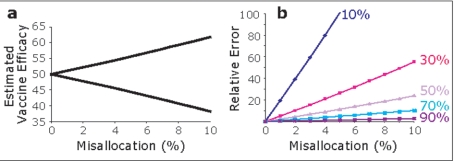
Influence of *CCL3L1-CCR5* GRG status on vaccine efficacy. (a) Estimated vaccine efficacy as a function of the percentage of subjects that are misallocated (m) with respect to their *CCL3L1-CCR5* genotype across the placebo or vaccine trial arms. The model is for a preventive vaccine that has a true efficacy of 50%. The upper line in this plot depicts estimated vaccine efficacy as a function of misallocation of subjects with a low GRG towards the vaccine arm, resulting in a fallacious increase in the estimated vaccine efficacy. The lower line depicts the converse situation, i.e., misallocation of subjects with a low GRG towards the placebo arm, resulting in a fallacious decrease in the estimated vaccine efficacy. (b) Plots depict the difference (relative error) between the true and estimated vaccine efficacy as a percentage of the true vaccine efficacy for varying values of m. Methods are described in SOM, section 2.

We considered that failure to account for the transmission-influencing effects of *CCL3L1-CCR5* genotypes might mask true efficacy estimates of a vaccine that partially blocks transmission (**SOM**, **section 2**). **Figure 3a** models the effects of misallocating subjects with respect to their *CCL3L1-CCR5* GRG status in the placebo or vaccine arms of a trial with a preventive vaccine which has an efficacy of 50%. This mathematical model is relatively impervious to HIV incidence and trial size number; and it predicted that, depending on its direction, misallocation will result in the over- or underestimation of the vaccine's true efficacy. For example, in a trial of 500 subjects, for a vaccine that has a 50% efficacy, misallocation of only 25 (5%) subjects has an estimated efficacy of 44% (95% confidence interval (CI) of 41%–45%), and this is a relative error of ∼12% (95% CI of 10%–18%) from the true vaccine estimate (**Figure 3a**). This effect of GRG-dependent misallocation is magnified when a vaccine with lower efficacy is considered in the model (**Figure 3b**). These effects of misallocation might be further compounded by the fact that vaccine-induced CMI might wane faster in subjects who possess the moderate and high GRGs.

## Discussion

The global distribution of HIV among populations is highly heterogeneous, even in a continent such as Africa [3], [30], [31], [32], [33], [34], [35], [36], [37], [38]. Many factors are likely to account for this heterogeneity, including viral factors, sexual behavior patterns, sexually transmitted infection, poverty, male circumcision status, unrest and wars, urbanization, and other social and economic reasons. However, the results of a large ecologic study indicated that the social, behavioral, economic and political confounders cannot in themselves account for this heterogeneity [32]. For example, sexual behaviors between cities in Africa with a high and low prevalence of HIV were similar [33], [34]. Additionally, despite extensive poverty, war and breakdown of health care systems, the steady-state prevalence of HIV in the Democratic Republic of Congo, a country near the epicenter of HIV, has remained remarkably stable and much lower than that of its neighboring nations [37], [38]. Thus, we [13], [16], [20], [70] and others [18], [24], [26], [32], [71], [72], [73], [74], [75] have proposed that in addition to viral and other factors, biologic factors such as the genetic make-up of populations might contribute to this heterogeneity in the global distribution of HIV. One of these previous studies used mathematical modeling to substantiate this possibility [71]; however, the analysis was confined to the contribution of the *CCR5-Δ32* mutation, which is restricted to individuals of European descent. By contrast, we have modeled the effects of a wider range of polymorphisms in *CCR5* and the copy number of *CCL3L1* that are determinants of HIV-AIDS susceptibility.

Our findings indicated that under the umbrella of an overall trajectory of the simulated HIV epidemic (**Figure 2c**, inset), the *CCL3L1-CCR5* GRG-defined partner pair population groups discriminate for several distinct subtrajectories with different shapes and time-scales (**Figure 2c**). Of these subtrajectories, only a few appear to be critical in sustaining the epidemic (**Figure 2c**). Conversely, these data suggest that human populations that are enriched for low GRGs, i.e., a high *CCL3L1* gene dose and nondetrimental *CCR5* genotypes, might be relatively protected against the spread of HIV-1. Bolstering this possibility, we recently found that HIV-infected individuals who maintained very low viral loads and who were disease free despite not receiving therapy (elite or viremic controllers of HIV) [76] were selectively enriched for a low GRG [53]. Given the importance of the VL in transmission, it is likely that such subjects will transmit virus at very low rates. Thus, the data indicating that *Ro* is lowest in HIV+/HIV− partner pairs in which the transmitting partner has a low-risk GRG status suggest that vaccines directed towards reducing infectivity of the HIV+ host may play an important role in halting epidemic spread. Underscoring this, findings from animal studies [67] and mathematical modeling [1], [2], [68], [69] support the hope that imperfect, T-cell based disease-modifying, i.e., therapeutic vaccines, by reducing plasma viral load at the population level might abate the epidemic.

**Figure 4:**
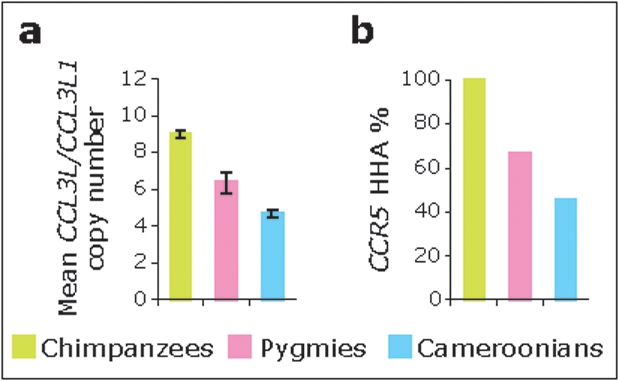
*CCL3L1* copy number (a) and *CCR5* HHA haplotype frequency (b) in Pygmies (N = 51) and Cameroonians (N = 372). The prevalence of the chimpanzee *CCR5* HHA haplotype and the ortholog of human *CCL3L1* in chimpanzee designated as *CCL3L* [20], is also shown (N = 83). Error bars in panel a denote 95% confidence interval.

Thus, the results of our modeling data indicate that there might be a *CCL3L1-CCR5*-dependent biological basis for interpopulation differences in HIV prevalence. We illustrate this concept further with the example of Pygmies, a distinct population in Central Africa. The high number of HIV-1 subtypes cocirculating, the high intrasubtype diversity, and the high numbers of possible recombinant viruses as well as different unclassified HIV strains are all in agreement with an old and mature epidemic in Central Africa, and suggest that these regions are the epicenter of HIV-1 [77], [78], [79]. The cross-species transmission of simian immunodeficiency virus (SIV)cpz to humans is now thought to have occurred by exposure to the blood of chimpanzees infected with SIVcpz during hunting and butchering of these primates in Central Africa [78], [79]. The Pygmies, one of the oldest ethnic groups in this area, have lived in Central Africa for more than 20,000 years. These hunter-gatherers have been, and continue to be, frequently and directly exposed to nonhuman primate blood during hunting, slaughtering and cooking. However, surprisingly, HIV/SIV infection in Pygmies is rare and occurs mainly after contact with Bantus rather than from contact with nonhuman primates ([80], [81] and references therein). The rarity of HIV infection via cross-species transmission from chimpanzee to Pygmies contrasts with the fact that documented zoonosis of other viruses has occurred into this population. Data indicates that human T-lymphotropic virus (HTLV)-1 diversity appears to have resulted from multiple cross-species transmissions of simian T-lymphotropic virus (STLVs)-1 following contract between humans and non-human primate species infected with STLV species [82]. Strikingly, Pygmies harbor a heterogeneous HTLV-1 strain, which is very similar to the STLV-1 in chimpanzees (STLV-1cpz) [83]. Might *CCL3L1-CCR5* genetic constitution help explain in part why cross-species transmission of HIV-1 has been rarely observed in Pygmies despite evidence of other viral zoonosis? In this respect, it is noteworthy that compared to other African populations that reside in geographical proximity (e.g., non-Pygmy Cameroonians), Pygmies have an even higher frequency of the protective ancestral *CCR5* haplotype designated as HHA [13], [16], [84] and the gene dose of *CCL3L1* is highest in this isolated population (**Figure 4**). Strikingly, all chimpanzees possess the protective ancestral *CCR5* haplotype HHA [13], [84], [85] and a high *CCL3L* copy number (**Figure 4**) [20]. Based on these observations, we conjecture whether this distinct *CCL3L1-CCR5* genetic makeup of Pygmies might explain the observed relative resistance to cross-species transfer of HIV-1 but susceptibility to other viral infections, as well as the lower prevalence of HIV relative to those of neighboring populations. By analogy, the *CCL3L1* and *CCR5* genetic makeup of chimpanzee might afford this nonhuman primate species protection against disease induced by natural SIV infection as well as to experimental infection with HIV [86]. The strikingly monomorphic host *CCL3L1-CCR5* genetic constitution of Pygmies contrasts with the extensive viral heterogeneity prevalent in Central Africa.

The findings in Figure 2 collectively suggest that the *CCL3L1-CCR5* GRGs play a role in determining not only the dynamics of the HIV epidemic but also in the design of prevention programs. We found that *Pc* was >1 in several HIV+/HIV− partner pairs defined by their *CCL3L1-CCR5* genotype and that a HIV vaccine with an efficacy of lower than 70% might be ineffective in controlling the spread of HIV in these genetically-defined population groups. This threshold of 70% is also relevant in the context of three prior observations. First, vaccine acceptability might be highest when the efficacy is at least 70% [87]; second, studies that have modeled the effects of a live-attenuated HIV vaccine have shown that efficacy is best matched with safety around this same threshold [88]; and third, most non-HIV vaccines in use today have an efficacy between 70 and 90% [89].

Our models consider the possible impact of the *CCL3L1-CCR5* GRGs on the evaluation of efficacy estimates of a sterilizing or prophylactic HIV vaccine. First-generation prophylactic HIV-1 vaccines are unlikely to provide complete protection from infection [41]. Consequently, there is a thrust to evaluate candidate imperfect HIV/AIDS vaccines, including those that can stimulate cell-mediated immune responses that are directed at controlling viral replication after acquisition of infection [41], [90], [91], [92]. Several of these vaccines are being evaluated in humans (http://www.iavi.org/). In addition to HIV acquisition, surrogate endpoints currently under consideration for assessment of such disease-modifying vaccines can be categorized as (a) immunologic, e.g., maintenance of the CD4+ T-cell counts; (b) virologic, e.g., decreased VL set-point; (c) clinical, e.g., reduced numbers of HIV-infected vaccinated subjects requiring antiretroviral treatment and/or developing clinical disease; and (d) epidemiological, e.g., lower sexual transmission rate by vaccinated subjects who become HIV-infected subsequent to vaccination [6], [93]. However, variations in *CCL3L1-CCR5* by impacting on (i) risk of acquiring HIV infection, (ii) viral load, (iii) rate and extent of CD4^+^ T cell depletion, and (iii) time from seroconversion to thresholds of CD4^+^ T cell counts at which antiviral therapy is initiated might confound the assessment of the currently used surrogates of vaccine endpoints.

Additionally, the finding that misallocation of subjects based on their genetic constitution, modeled here according to *CCL3L1-CCR5* genotypes, might result in fallacious estimates of a vaccine's efficacy, has relevance for several reasons. First, it is possible that confounding due biological factors may influence both the evaluation and outcome of a vaccine [94]. This is best illustrated by the results of the STEP trial where antibody titers to Adenovirus 5 appear to have confounded the outcome and the interpretation of the efficacy of the virus [39], [40], [41], [42], [43], [44]. We have suggested that these higher titers could be due to an underlying host genetic constitution that may convey a “stronger immune system” [40]. Additionally, after the failure of two vaccine trials there is a need to rapidly evaluate vaccine candidates, especially in smaller numbers of individuals before conducting larger and more expensive trials. Although our modeling is based on a randomized trial which is impervious to HIV incidence and the number of trial participants, the confounding due to misallocation of favorable or unfavorable genotypes in trial arms may be important in three scenarios – trials with small sample sizes; interim analysis of large trials; and trials that demonstrate equivalence between two candidate vaccines [95]. At least in these situations the baseline *CCL3L1-CCR5* genotyping of the trial participants might have relevance so as to either resort to a stratified randomization protocol or to post-allocation adjustment for the potential influence of the genotypes on vaccine efficacy. However it is also important to note that large vaccine evaluation trials (e.g., recent STEP trial) are invariably a composite of subjects from several different geographically disparate clinical sites, and consequently, failure to account for *CCL3L1-CCR5* genotype at these smaller sites in subjects with different racial backgrounds might result in confounding of a larger multicenter trial. This might be an important point to consider in light of two observations. First, the *CCR5* genotypes that influence AIDS susceptibility differ according to ethnicity [13], [18]. Second, the high-risk HIV-negative cohorts likely to be recruited for vaccine trials might be enriched for genotypes that confer protection. Hence, if a vaccine were to be tested in such subjects it might be difficult to differentiate between the protective effects of the HIV vaccine versus those of *CCL3L1-CCR5* genotype or other protective genotypes. Two observations support this possibility: (i) in a cohort of high-risk female sex workers from South Africa, we found an enrichment of subjects with high copy numbers of *CCL3L-* and *CCL4L-*containing segmental duplications (Ramsuran et al, unpublished data); and (ii) a previous study found that there is an enrichment over time of protective *HLA* genotypes in a cohort of East African female sex workers [96].

Thus, taken together, our findings suggest that the inherent variability among individuals and, by extension, among populations, in host genes that influence HIV-AIDS susceptibility might be an important but hitherto underestimated biological challenge to contend with in the quest for an effective HIV-AIDS vaccine. Extending the notion of pharmacogenomics, which links host genetic determinants to variable responses to pharmacologic agents, we suggest that vaccinogenomics is an equally important concept to consider in the design and evaluation of vaccines, not only for established scourges such as HIV-1, tuberculosis, and malaria, but also for emerging infectious diseases.

## Materials and Methods

Our modeling studies used the *CCL3L1-CCR5* genotyping data from the HIV-positive subjects from the Department of Defense (DoD) HIV Natural History Study (NHS) cohort followed at Wilford Hall Medical Center (WHMC) and more recently at the Brooke Army Medical Center (BAMC), San Antonio, TX. The studied population is the local component of a prospective multisite observational cohort from the United States Military's Tri-Service AIDS Clinical Consortium (TACC) HIV Natural History Study. Extensive details of this cohort has been provided elsewhere [13], [20], [53]. Unidentified cast-off blood from subjects participating in training at Lackland AFB, TX was used for the HIV-negative control population. The definitions of all the model parameters are shown in **Table S1**.

### *Pc* estimate

For our analyses, we used the conceptual and mathematical frameworks developed previously for the epidemiological context of vaccination against HIV-AIDS [1], [2]. These models rely on computing the *Pc*, which is extensively used as an estimate of the critical proportion of the population- or cohort-based vaccination coverage required to limit an epidemic. This estimate has three main components (*Ro*, *e* and *f*) which are shown in the equation below.

*Pc* = [1-(1/Ro)]/ef. Thus, *Pc* is a function of i) *Ro*, the basic reproduction number which provides a measure of the average number of secondary infections generated by one primary case of infection in a susceptible population; ii) *e*, the vaccine efficacy; and iii) *f*, the fraction of vaccinated subjects in whom the vaccine effect does not wane over the period of infectiousness, i.e., the duration of protection afforded by the vaccine. These three parameters are shown in Figure 1b, and we have termed *f* in this figure as vaccine durability. The relationship among *Ro*, *e* and *Pc* for a fixed *f* is shown in the figure in **Supplementary Online Materials S1 (SOM)**, **Section 1.1**.

We used a deterministic, compartmentalized mathematical model (Figure 1) to assess the influence of the *CCL3L1-CCR5* GRGs on the population dynamics of HIV infection. The dynamics were based on the estimation of the reproductive number, *R_o_*. *R_o_* was computed within each *CCL3L1-CCR5* GRG-defined population strata as well as for the overall population, and detailed methods employed and the assumptions underlying the mathematical model are provided in the **SOM**, **Section 1.1 and 1.2**. We accounted for the possible effect of the *CCL3L1-CCR5* GRGs on vaccine efficacy and durability as discussed in **SOM**, **sections 1.3 and 1.4**, respectively. To ensure generalizability of the results of our modeling studies we also conducted sensitivity analyses on the model parameters, the results of which are shown in **SOM**, **section 1.5**. Attributable fraction and critical response time were calculated as described in **SOM**, **sections 1.6 and 1.7**, **respectively**. In our mathematical modeling, we assumed that the risk behavior and probability of circumcision is not influenced by *CCL3L1-CCR5* GRG status.

### Influence of the unequal distribution of *CCL3L1* and *CCR5* genotypes in trial arms on the estimates of vaccine efficacy in preventive trials

We simulated a typical randomized two-arm trial design to examine the influence of the genotypic imbalance across trial arms on the estimates of HIV vaccine efficacy. The full derivation of the mathematical model used for estimating this influence is provided in the **SOM**, **section 2**.

## Supporting Information

Supplementary Online Material S1 Role of CCL3L1-CCR5 genotypes in the epidemic spread of HIV-1 and evaluation of vaccine efficac.
(0.57 MB DOC)

Table S1 Summary of parameters used to model the influence of CCL3L1-CCR5 GRGs on epidemiological endpoints. Note, the inclusion of the parameters of vaccine efficacy and durability were included on a proof-of-principle basis for vaccines which rely in part on CMI for their effects.
(0.03 MB DOC)

Table S2 Group, indicates subdivision of the population into 9 groups based on their GRGs (Figure 2a). Estimated frequency indicates the proportion of the population groups from data derived from the WHMC cohort. The parameters are described in Supplementary Table S1.
(0.05 MB DOC)
