# Supplementary material for: Role of CCL3L1-CCR5 Genotypes in the Epidemic Spread of HIV-1 and Evaluation of Vaccine Efficacy
Source: PLoS One. 2008 Nov 7;3(11):e3671. doi: 10.1371/journal.pone.0003671 (PMC2576446; doi:10.1371/journal.pone.0003671)
Supplement: Supplementary Online Material S1 — Role of CCL3L1-CCR5 genotypes in the epidemic spread of HIV-1 and evaluation of vaccine efficac. (0.57 MB DOC) [file pone.0003671.s001.doc]

**SUPPLEMENTARY ONLINE MATERIAL FOR**

**Role of *CCL3L1-CCR5* genotypes in the epidemic spread of HIV-1 and evaluation of vaccine efficacy**

**Hemant Kulkarni**^1^**, Vincent C. Marconi**^2,3,4^**, Brian K. Agan**^2^**, Carole McArthur**^5^**, George Crawford**^1^**, Robert A. Clark**^1^**, Matthew J. Dolan**^3,4,6^**, and Sunil K. Ahuja**^1,7^

^1^Veterans Administration Research Center for AIDS and HIV-1 Infection, South Texas Veterans Health Care System, San Antonio, Texas 78229, USA. and Department of Medicine, ^7^Microbiology and Immunology and Biochemistry, University of Texas Health Science Center, San Antonio, Texas 78229, USA. ^2^Infectious Disease Clinical Research Program, Uniformed Services University of the Health Sciences, Bethesda, Maryland 20814, USA. ^3^Infectious Disease Service and ^6^Henry M. Jackson Foundation, Wilford Hall United States Air Force Medical Center, Lackland Air Force Base, Texas 78236, USA. ^4^San Antonio Military Medical Center, Fort Sam Houston, Texas 78234, USA. ^5^Department of Oral Biology, School of Dentistry, University of Missouri-Kansas City, MO 64108, USA

Table of Contents

| Section | Title | Page |
| --- | --- | --- |
| 1 | Mathematical modeling of the influence of *CCL3L1-CCR5* GRG status on epidemiological endpoints such as *Pc*, HIV spread and vaccine efficacy | 3 |
|  | 1.1 The *Pc* estimate | 3 |
|  | 1.2 The *Ro* estimate | 5 |
|  | 1.3 Vaccine efficacy (*e*) | 9 |
|  | 1.4 Vaccine durability (*f*) | 11 |
|  | 1.5 Relative importance of *Ro*, *t* and *f* – Sensitivity analyses | 12 |
|  | 1.6 Epidemic burden explained by the GRGs- Attributable fraction (AF) | 13 |
|  | 1.7 Critical response time (CRT) | 14 |
|  | 1.8 Risk behavior and circumcision | 14 |
|  | 1.9 Predicted epidemic trajectories within population strata | 14 |
| 2 | Influence of the unequal distribution of *CCL3L1* and *CCR5* genotypes in trial arms on the estimates of vaccine efficacy in preventive trials | 16 |
|  | References | 21 |
|  |  |  |
|  |  |  |
|  |  |  |

**Section 1. Mathematical modeling of the influence of *CCL3L1-CCR5* GRG status on epidemiological endpoints such as *Pc*, HIV spread and vaccine efficacy**

**1.1 The *Pc* estimate**

For these analyses, we used the conceptual and mathematical frameworks developed previously for the epidemiological context of vaccination against HIV/AIDS [1,2]. These models rely on computing the *Pc*, which is extensively used as an estimate of the critical proportion of the population- or cohort-based vaccination coverage required to limit an epidemic. This estimate has three main components (*Ro*, *e* and *f*) which are shown in the equation below.

*Pc* = [1-(1/Ro)]/ef (1)

Thus, *Pc* is a function of i) *Ro*, the basic reproduction number which provides a measure of the average number of secondary infections generated by one primary case of infection in a susceptible population; ii) *e*, the vaccine efficacy; and iii) *f*, the fraction of vaccinated subjects in whom the vaccine effect does not wane over the period of infectiousness, i.e., the duration of protection afforded by the vaccine. These three parameters are shown in Figure 1b, and we have termed *f* in this figure as vaccine durability. The relationship among *Ro*, *e* and *Pc* for a fixed *f* is shown in the figure below.


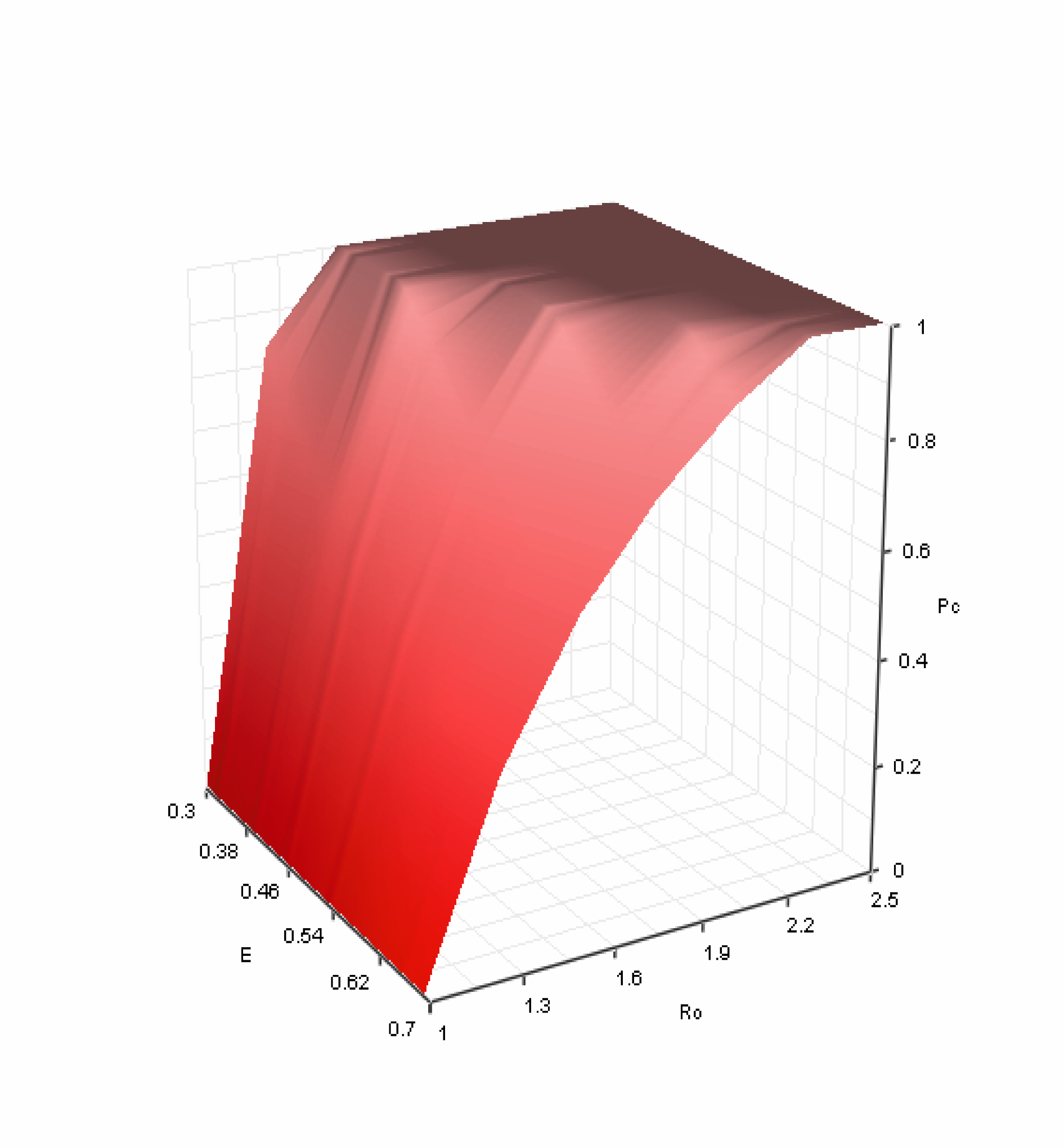


**Relationship between basic reproductive number, vaccine efficacy and the critical proportion needed to be vaccinated to limit the epidemic.** This is a surface plot showing the relationship between the vaccine efficacy (*e*), the basic reproductive number (*Ro*) and the critical proportion of cohort (*Pc*) that is needed to be vaccinated for eradication of the HIV epidemic. The surface describes the relationship *Pc* = [1-(1/Ro)]/ef as described by Blower et al [1] where, *f* represents the proportion of vaccinated subjects in whom the vaccine maintains its protective effect during the time the subject acquires new sexual contacts. For this curve, we assumed *f* to have a value of 0.8 as suggested by Blower et al [1].

We approached modeling of the influence of GRGs on *Pc* with the notion that

(i) these are proof-of-principle studies with the modeling conducted based on data derived from portions of the HIV^+^ DoD HIV Natural History Study cohort and cast-off blood specimens from HIV-negative USAF trainees [3,4]. Detailed characteristics of the U.S.-DoD HIV NHS cohort have been described previously [3,4,5].

(ii) *CCL3L1-CCR5* genetic risk groups (GRGs) will influence the *Pc* primarily by influencing infectiousness and susceptibility, and duration of the infectiousness (time-from-HIV acquisition to time-to-AIDS). In addition, we considered the possibility that the GRGs by affecting cell-mediated immunity (CMI) might also affect the take and durability of vaccines that rely on generation of cellular responses (see below). The GRGs are designated as low, moderate and high [4].

The methods we used to determine to calculate *Ro*, *e* and *f*, and thus *Pc* after accounting for the effects of the GRGs are discussed below, and the definitions of the various parameters studied herein are shown in Supplementary Table S1.

**1.2. The *Ro* estimate**

It is assumed that the transmission probability (ß), background death rate (μ) and proportion of the HIV-infected subjects progressing annually to AIDS (σ) together determine the parameter designated as the basic reproductive number (*Ro*) in the following manner:

*Ro* = ß/(μ + σ) (2)

The parameter *Ro* is of great interest in predicting the epidemic behavior or trajectory of HIV since it captures the number of secondary cases per unit time. Thus, *Ro* is a measure of the product of infectiousness and susceptibility, which are important determinants of the threshold or tipping point of the epidemic. A *Ro* estimate that exceeds unity favors an epidemic whereas a value lower than unity favors conditions that will limit the epidemic. When estimating *Ro*, we assumed a background death rate of 0.025 (as suggested by Anderson and Hanson [2]) and a σ of 0.043. We estimated σ using data from the seroconverting component of the HIV^+^ WHMC cohort. Since the denominator in equation 2 can be assumed to be constant across the GRGs, in essence, the behavior or trajectory of the epidemic will be determined by the transmission probability (ß).

Based on our previous findings [3,4,6], we surmised that the transmission probability (ß) should vary by the GRGs, and to calculate this parameter we did the following. After considering the GRGs of the partners in a heterosexual setting of HIV transmission, the population was divided into nine groups (shown in Figure 2a and Supplementary Table S2). To calculate *Ro* in each of these nine groups, the following assumptions were made regarding the *infected partner*.

1. GRGs can influence the initial viral load (VL). This assumption is based on the results of our prior findings (e.g. Figure 2b in Dolan et al [4]).
2. The VL setpoint can determine the degree of infectiousness [7,8]. For example, Gray et al have demonstrated that the per sexual contact probability of HIV transmission can be estimated based on the viral load [8]. They showed that a reduction in log viral RNA from 4.58 to 3.23 was associated with a 23-fold decrease in the transmission probability.
3. We assumed that the duration of infectiousness as the duration from time-of-seroconversion to time-of-development of AIDS. Thus, the rate of disease progression to AIDS will influence the duration of infectiousness.
4. Previously [3,4] we had shown that the GRGs influence the rate of disease progression by influencing VLs. However, we also found that *over and above* their influence on the VL setpoints, GRGs can independently alter the duration of infectiousness [4]. This is based, for example, on our prior findings where we found that after adjusting for the VL, baseline CD4^+^ T cell counts and several other parameters that are known to influence AIDS progression rates, the GRGs independently affected the rate of disease progression (Table 2 in Dolan et al [4]).

To calculate *Ro* in each of these nine groups, the following assumptions were made regarding the *uninfected partner*. Based on findings shown in Figure 4 (H and I) in Gonzalez et al [3] which showed that *CCL3L1-CCR5* genotype influence risk of HIV acquisition in adults and in the setting of vertical transmission, we assumed that GRGs will influence susceptibility in the uninfected partners within the nine population groups.

With these assumptions in mind, we calculated the *Ro* in each of these nine groups as follows.

1. We first factored in the effect that the GRGs will have on the probability of transmission from the infected partner by virtue of their effects on the VL setpoint. This is supported by findings showing that higher VLs is among the principal determinants of heterosexual transmission [7,8,9,10,11,12]. We calculated the annual probability of transmission from the infected partner as a function of the influence of the GRGs on the VL setpoint, and this parameter is designated here as ß_u_. To calculate ß_u_, we first estimated the mean log HIV RNA load within the three GRGs (derived from data published in [3]). Then using the equation provided by Gray et al [8], we estimated the per sexual contact probability of HIV transmission for each of the GRGs. Then, assuming an average coital frequency every two days, we estimated the annual transmission probability for each GRG. The ß_u_ for the nine population groups is shown in Supplementary Table S2.
2. To factor in the effect that the GRGs will have on the duration and degree of infectiousness together, we next calculated a parameter that we designate as ß_i_. ß_i_ takes into account both the disease-accelerating effects of GRGs *independent* of VLs (this is a measure of the duration of infectiousness) and the effects of GRGs on VL, i.e., ß_u_ (degree of infectiousness). We used the adjusted RHs shown in Table 2, model 13 in Dolan et al [4] for the three GRGs as a measure of the duration of infectiousness that is attributable directly to the GRGs. We used these RHs as they reflected the rate of disease progression to AIDS associated with the GRGs that is independent of the VL. ß_i_ is the product of the adjusted RHs and the ß_u_. Note, the effects of VLs on duration of infectiousness are not considered here because their effects have been incorporated into ß_u_.
3. We next factored in the effect that the GRGs will have on susceptibility of the uninfected partner, and for this we calculated a parameter that we designate as ß_a_. To estimate the probability of transmission in a specific population group (ß_a_) we factored in both the transmission probability ß_i_ as obtained in (ii) and the odds ratio (OR) of HIV-acquisition based on the susceptible partner’s GRG. Based on data in Gonzalez et al [3] we found that the ORs of HIV-acquisition were 1.00, 1.62 and 2.23 in the low, moderate and high GRGs in HIV-negative adults (the three GRGs are those designated in Figure 1a of this manuscript). Thus if ß is the probability of transmission from an infected partner then the probability of transmission to the susceptible partner will be dictated by the OR associated with the GRG of the susceptible partner in the following way: ß_a_ = ß_i_OR/[1+(OR-1)ß_i_]. The values for ß_a_ are shown in Supplementary Table S2.
4. Finally, to obtain *Ro*, we factored in the background death rate and annual incidence rate of AIDS in the HIV-infected subjects into the ß_a_ for each of the nine population groups. For this, we divided the transmission probability ß_a_ by the sum of background death rate and annual incidence rate of AIDS in HIV-infected subjects (μ and σ) to obtain the population group-specific estimate of *Ro* (Supplementary Table S2).
5. These calculations further assume that even though the studied cohort is a predominantly male cohort (~94%) the results are applicable to the general population and that the transmission probabilities are only minimally affected by the gender.
6. Lastly, as the data is derived from a U.S-based cohort (WHMC), these calculations will be most directly applicable to clade B HIV-1 infections that are prevalent in the U.S. However, the conceptual basis of the model is also applicable to other geographic settings.
7. We also assumed random mixing of the sexual partners.

**1.3. Vaccine efficacy (*e*)**

Vaccine efficacy is composed of two components: vaccine *take* and *degree* [1], designated as *t* and *d*, respectively in Figure 1b. As defined by Blower and colleagues [1], *take* specifies the fraction of vaccinated individuals that show a protective immunological response to the vaccine. *Degree* specifies the degree of vaccine-induced protection against HIV infection experienced by the individuals in which the vaccine takes.

Because of their effects on immune responses, it is possible that variations in *CCL3L1* and *CCR5* could possibly affect both the humoral and cellular immune responses elicited by vaccines. We do not have access to cohorts to determine the relationship between *CCL3L1-CCR5* variations and humoral responses. However, based on their influence on CMI responses shown in Dolan et al. [4] , the GRGs can be thought to influence both the *take* and *degree* of vaccines that require elicitation of cell-mediated immune responses. In our mathematical modeling, we therefore modeled this possible effect of the GRGs on CMI in the *Pc* estimate. In these proof-of-principle studies, we considered this to be relevant because several vaccines that rely on CMI for its effects are being considered for development and testing. More importantly, we sought to conduct these analyses to determine the relative importance of *Ro* versus the importance of vaccine efficacy/durability on the *Pc* estimate.

For simplicity here we assumed the *degree* to be constant across GRGs. Additionally, as a conservative measure of the influence of GRGs on the initial vaccine ‘take’, we estimated the relative vaccine take (parameter *t* in Supplementary Tables S1 and S2 and Figure 1b, this study) by normalizing the best delayed type hypersensitivity (DTH) skin test responses for each GRG. We used DTH responses as a surrogate marker for cell-mediated immunity (CMI), and demonstrated that *CCL3L1/CCR5* genotypes affect CMI in both HIV-seronegative and HIV-seropositive individuals [4]. We found that the average number of the best positive skin tests was 2.77, 2.60 and 2.37 in the low, moderate and high risk GRGs, respectively [4]. If we normalize the average number of positive skin tests in the low-risk group to 100%, then the relative vaccine take in the three GRGs (as shown in the adjoining figure) can be estimated as 100%, 94% (95% CI 88% - 100%) and 86% (95% CI
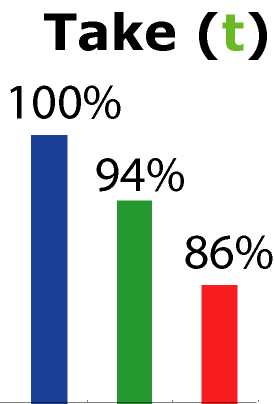
74% - 98%). We stress, that these parameters would be for the effects of the GRGs on a vaccine that relies, in part, on the generation of robust CMI responses.

These percentages appear to be relatively conservative based on the known variability of DTH responses in normal individuals. For example, a study of DTH responses in normal Australians showed that 3% and 5.6% of men and women, respectively were anergic (no positive responses), and 10.6% and 9.4% of men and women, respectively fell into the "hypoergic" category[13].

**1.4. Vaccine durability (*f*)**

With the same caveats outlined above, we also modeled the influence of the GRGs on the duration of protection of a vaccine that relied in part on generation of CMI. Vaccine durability has been referred to as *f* by Blower et al [1]. Results in Dolan et al.[4] suggested that in HIV-infected subjects DTH responses decline over time and that the degree of this decline might be a function of the GRGs. In turn, this suggests that the duration of vaccine protection can also vary across GRGs. We sought to use this information as a means to model waning vaccine durability over time as a function of the GRGs. In accord with Anderson and Hanson [2], we assumed that the duration of vaccine protection will be 10 years, and as such we assumed that this duration of vaccine durability for the low GRG. This translates to an annual probability of a waning in the vaccine effect of 0.1.


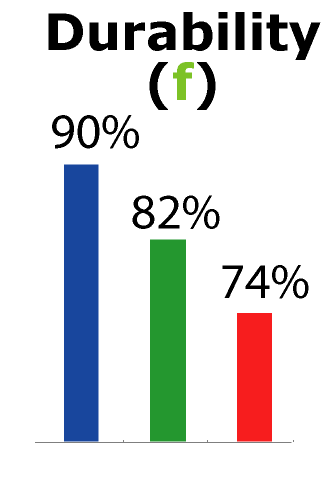
In the next step, we estimated the risk of anergy (complete absence of DTH response) across GRGs. We observed that compared to possession of the low GRG, possession of the moderate and high GRGs was associated with an increased likelihood of anergy. The odds ratio for anergy was 1.91 (95% CI 1.07 – 3.39, *P* = 0.028) in the moderate GRG and 3.10 (95% CI 1.36 – 7.05, P = 0.007) in the high GRG. Using these estimates of odds ratios, we determined that the annual probability of the loss of vaccine effect will be 0.1, 0.18 and 0.26 in the low-, moderate- and high-risk GRGs, respectively. As vaccine durability = 1/annual probability of loss of vaccine effect, these estimates translate to a vaccine durability of 10 yrs, 5.6 yrs and 3.9 yrs, respectively in the low, moderate and high GRGs. In other words, 90%, 82% (95% CI 73% - 88%) and 74% (95% CI 56% - 87%) of the vaccinated subjects can be expected to annually not fail the vaccine (adjoining figure).

**1.5. Relative importance of *Ro*, *t* and *f* – Sensitivity analyses**

Since (i) our estimates of *Ro, t* and *f* were projections and (ii) in our models we did not include the potential effects of a preventive vaccine via the humoral arm of the immune system, we assessed the relative importance of these parameters on the estimate of *Pc*. To this end, we conducted one-way sensitivity analyses. We assumed the following baseline (range) values for these parameters: *Ro*, 2.0 (1.0 – 10.0); *t*, 0.8 (0.6 – 1.0) and *f*, 0.9 (0.6 – 1.0). Using these values we conducted sensitivity analyses, the results of which are shown in the tornado diagram below.


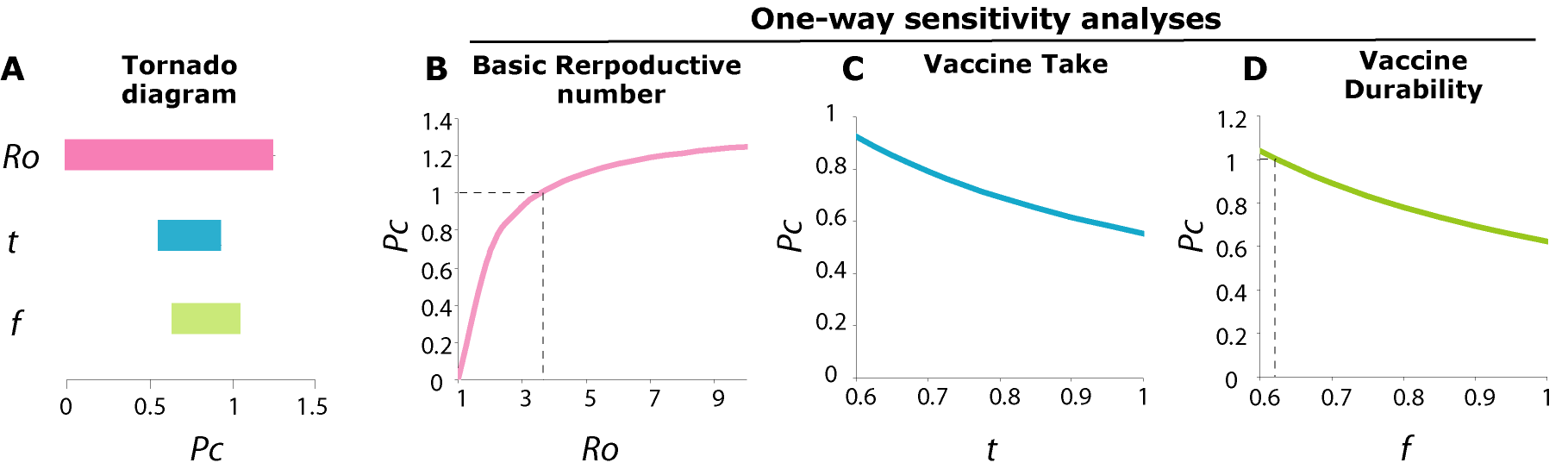


**Results of one-way sensitivity analyses of the components of *Pc*.** (**A**) Tornado diagram. The bars represent the extent of variation in *Pc* in response to a variation in each of the components – *Ro*, *t* and *f* – within the range specified above. The *Pc* is most sensitive to changes in *Ro*. (**B** to **D**) One way sensitivity analyses for each component was conducted by fixing the remaining components to the baseline values. The dashed lines indicate the point where mass vaccination (*Pc* = 1) is required.

The results demonstrate that over the selected range, the *Pc* estimate is most sensitive to the *Ro* as indicated by the widest bar in the tornado diagram. In one way sensitivity analysis, when we fixed the baseline values of *t* and *f*, we observed that a *Ro* greater than 3.57 leads to a *Pc* value exceeding unity and this might imply the need for mass repeated vaccinations or use of a more efficacious vaccine. By contrast, for the baseline value of *Ro* and *f*, variation of *t* over the indicated range did not entail a need of vaccination while for the baseline values of *Ro* and *t*, a value of ≤ 0.625 for *f* suggested a need for mass vaccination. Considering the values of *t* and *f* used in our analyses (Supplementary Table S2) it is evident that *Ro* is the major critical determinant of *Pc*.

**1.6. Epidemic burden explained by the GRGs- Attributable fraction (AF)**

In the epidemiologic literature AF is commonly used to estimate the burden of disease that can be attributed to the presence of a putative risk factor. In accord with this concept, we estimated the burden of the projected epidemic that is attributable to the GRGs of the infected and the susceptible partners in the target population. Since *Ro* is the critical parameter dictating the epidemic behavior for a fixed value of *t* and *f*, we used the estimated value of *Ro* as a measure of the strength of association between the population stratum (determined on the basis of the GRGs of infected and susceptible partners) and the severity of the potential epidemic in each of these population groups. Then, using the method of estimating AFs for a multiple category risk factor as described previously [14], we estimated the AF for the entire target population and for each stratum within the target population (Figure 2d, this study). For computing the AF, we used the estimated frequency of the population groups (Figure 2a, this study) and the *Ro* (Figure 2b, this study).

**1.7. Critical response time (CRT)**

Another outcome of public health interest in epidemics is the critical response time, defined as the minimum available time for planning and implementing the preventive public health actions so as to prevent an impending epidemic [15]. This response time is inversely proportional to the probability of transmission and is estimated as 1/ß. We estimated the CRT (Figure 2e) within each stratum of the target population based on the estimated probability of HIV-1 transmission described in section 1.2.

**1.8. Risk behavior and circumcision**

In our mathematical modeling, we assumed that the risk behavior and probability of circumcision is not influenced by the GRGs.

**1.9. Predicted epidemic trajectories within population strata.**

Having estimated the *Ro* within each population stratum defined by the *CCL3L1-CCR5* GRGs of the infected and susceptible partner, we conducted proof-of-principle studies to determine the effects of the GRGs on the epidemic trajectories. For this purpose, we predicted the epidemic trajectory within each population stratum defined by the GRGs; based on the GRGs of the HIV-positive index partner and susceptible partner, the population can be subdivided into nine strata. A discrete-time, compartmental, susceptible-infected-removed (SIR) model of epidemics was used [16,17,18]. As indicated above, the estimates of *Ro* were derived from annual probability of transmission. Therefore, we predicted the time course of the epidemic in years since the beginning of the epidemic. For this analysis, we assumed a closed and non-growing population and thus allowing the epidemic to die out naturally. Also, we assumed a relatively homogeneous population within each stratum. An initial population size of 1 million was assumed and the epidemic was assumed to have been initiated by a single index case.

To estimate the overall *Ro* in the target population, we used two complementary strategies as suggested by De Jong et al [19] and Hyman and Li [20]. In the first strategy, the overall *Ro* is defined as the spectral radius of the “next generation matrix”, R. The cell R_ij_ of the next generation matrix represents the expected number of secondary infections in unvaccinated people in subgroup i resulting from a single randomly selected unvaccinated infectious person in mixing group j. To obtain this next generation matrix, we first represented the information presented in Figure 2a and 2b (main text) in the following two matrices:

$RoMat=\left[\begin{matrix} 0.96 & 1.97 & 4.29 \\ 1.22 & 2.47 & 5.15 \\ 1.39 & 2.79 & 5.69 \end{matrix}\right]$ ,$F=\left[\begin{matrix} 0.2981 & 0.2482 & 0.0462 \\ 0.1795 & 0.1493 & 0.0278 \\ 0.0256 & 0.0213 & 0.0040 \end{matrix}\right]$

where, ***RoMat*** contains the *Ro* values and ***F*** contains the frequencies of the nine population groups listed in Figure 2a and 2b (this study). Using the notation of Hill and Longini [21], the next generation matrix, R, can be derived as a matrix multiplication of ***RoMat*** and ***F***. Thus,

$$R=\left[\begin{matrix} 0.75 & 0.62 & 0.12 \\ 0.94 & 0.78 & 0.15 \\ 1.06 & 0.88 & 0.16 \end{matrix}\right]$$

Based on this next generation matrix, the overall *Ro* is defined as the spectral radius of the ***R*** matrix and is computed as the largest eigenvalue of the ***R*** matrix. In this case, the largest eigenvalue is 1.70 and thus, overall *Ro* was considered as 1.70.

Alternatively, Hyman and Li [20], define the overall *Ro* as a weighted average of the *Ro* values within each stratum. We used the subpopulation frequencies as the weights for estimating the average *Ro* which was 1.82. Thus, both strategies yielded comparable estimates of *Ro*. We used the latter estimate of the *Ro* to generate the overall epidemic trajectory shown as inset to Figure 2c.

**Section 2. Influence of the unequal distribution of *CCL3L1* and *CCR5* genotypes in trial arms on the estimates of vaccine efficacy in preventive trials**

Randomization is resorted to in clinical/preventive trials in an attempt to achieve a balanced distribution of the known and unknown confounding variables. We simulated a typical two-arm trial design to examine the influence of the genotypic imbalance across trial arms on the estimates of HIV vaccine efficacy. Our methods were as follows.

To start with, let us assume that the potential role of genotypes on the risk of acquisition of HIV as well as the distribution of the genotypes in the trial sample is not known. The table shown below summarizes the results from such a trial. Thus, the relative risk (r) of infection is defined as a/v ÷ c/u and the vaccine efficacy (ê) is estimated as 1-r. Now, let us assume that we know that the possession of a particular genotype increases the risk of acquiring HIV infection. In our case, for example, the possession of either the *CCR5* detrimental genotype or *CCL3L1* copy number lower than the population-specific median copies (or both) can increase the risk of HIV-acquisition 1.72-folds (95% CI 1.44 – 2.04, p = 8.8x10^-10^). If randomization is proper and adequate, then we expect that the proportion of vaccinees with the low-risk GRG will be the same as the proportion of unvaccinated subjects possessing the low risk GRG and the prevalence in the general population of the low-risk GRG (p_o_, in the case of WHMC cohort p_o_=0.5). When, however, partial misallocation occurs then the estimate of vaccine efficacy can be expected to be biased because of the unequal risk of acquiring HIV across GRGs and because of the unequal distribution of the genotypes across trial arms.

| Vaccine | Infection | | Total |
| --- | --- | --- | --- |
|  | Yes | No |  |
| Yes | a | b | v |
| No | c | d | u |
| Total | p | q | n |

Let I_u_ be the incidence of the HIV-infection in unvaccinated subjects and let I_v_ be incidence of HIV-infection in the vaccinees. If e is the true vaccine efficacy, then e = 1 – (I_v_/I_u_), and alternatively I_v_ = (1-e)I_u_. (1)

The incidence of infection occurring in the each trial arm can be considered as a weighted (based on the prevalence of respective GRGs) average of the risk of acquiring HIV-acquisition across the GRGs. Thus, in the unvaccinated subjects, if p_ou_ is the prevalence of the low-risk GRG, then the expected number of infected subjects at the end of the trial will be

nI_u_[p_ou_ + (1-p_ou_)r], (2)

where, n is the number of subjects recruited in the trial. Similarly, if p_ov_ is the prevalence of the low risk GRG in the vaccinated subjects, then the expected number of cases in the vaccinated group will be

nI_v_[p_ov_ + (1-p_ov_)r], (3)

Substituting the value of Iv from (1), we get

n(1-e)I_u_[p_ov_ + (1-p_ov_)r], (4)

The estimated vaccine efficacy (ê) can then be calculated as

, since n and I_u_ cancel out. (5)

If there is no misallocation, then p_ov_ = p_ou_ and ê = e.

Now, if *m* represents the fraction of the trial subjects misallocated so that there is an enrichment of subjects with high/moderate risk GRG in the vaccinated subjects at the cost of the low risk GRGs in the unvaccinated subjects and ρ represents the ratio of vaccinated to unvaccinated subjects enrolled in (and who completed) the study, then it can be shown that both p_ov_ and p_ou_ are functions of po, m and ρ and can be estimated as

(6)

and

(7)

In the cases of equal size of the trial arms, ρ=1 and p_ov_ = p_o_-2m while p_ou_ = p_o_+2m. In other words this means that the prevalence of the low-risk GRG is reduced in the vaccinated group and increased in the unvaccinated group by a factor proportional to the fraction of subjects misallocated. Consequently, one can expect an excess (than expected) of HIV-infections in the vaccinated group and a reduction (than expected) of HIV-infections in the unvaccinated subjects. This, in turn, can be expected to lead to a decreased estimate of the vaccine efficacy. If, we substitute equations (6) and (7) into equation (5), we get

(8)

This equation captures the direct relationship between the degree of misallocation (m) and the estimated vaccine efficacy (ê).

Using the estimates of p_o_ and r from the WHMC data, assuming a trial of equal sized arms (that is ρ = 1) and varying the true vaccine efficacy, we assessed the influence of the degree of misallocation (m) on the estimates of the vaccine efficacy that would have resulted from a trial with inadequate randomization. The results are shown in Figure 3a and Figure 3b (main text). Figure 3a shows the estimates of vaccine efficacy (ê) for varying values of m (shown as percentage) and a true vaccine efficacy of 50%. Since m is treated here as the proportion of subjects misallocated, this interpretation is essentially sample size invariant unless it can be assumed that the proportion misallocated is itself a function of the sample size. Figure 3b shows the difference between the true vaccine efficacy and the estimated vaccine efficacy as a percentage of the true vaccine efficacy for varying values of *m*.

We observed that the vaccine efficacy-reducing influence of misallocation was magnified if the true vaccine efficacy was low (Figure 3a). Based on the results shown in Figure 3b (main text), the relative error in estimates of vaccine efficacy can vary between 0.2% - 19% based on vaccine efficacy for a very small misallocation rate of 1%. It is expected that most of the candidate vaccines will have a partial protective effect. As an example, in a trial of a 50% efficacious vaccine on 500 subjects, misallocation of only 5% (25) subjects will lead to an estimate of 44% for vaccine efficacy (95% confidence interval 41% - 45%) – a relative error of ~12% in the estimate. Therefore, randomization based on genotypic information can be expected to ameliorate the confounding in the estimates of vaccine efficacy. As an alternative, stratified statistical analysis based on the genotypic information (e.g. Mantel-Haenszel test) can overcome the confounding in the estimates of vaccine efficacy. Therefore, whether at the time of recruitment or statistical analysis, knowledge of the GRGs of the study subjects will refine the estimates of vaccine efficacy.

Note, the inclusion of the parameters of vaccine efficacy and durability were included on a proof-of-principle basis for vaccines which rely in part on CMI for their effects.

|  |  |  |
| --- | --- | --- |
|  |  |  |
|  |  |  |
|  |  |  |
|  |  |  |
|  |  |  |
|  |  |  |
|  |  |  |

**References**

1. Blower S, Schwartz EJ, Mills J (2003) Forecasting the future of HIV epidemics: the impact of antiretroviral therapies & imperfect vaccines. AIDS Rev 5: 113-125.

2. Anderson R, Hanson M (2005) Potential public health impact of imperfect HIV type 1 vaccines. J Infect Dis 191 Suppl 1: S85-96.

3. Gonzalez E, Kulkarni H, Bolivar H, Mangano A, Sanchez R, et al. (2005) The influence of CCL3L1 gene-containing segmental duplications on HIV-1/AIDS susceptibility. Science 307: 1434-1440.

4. Dolan MJ, Kulkarni H, Camargo JF, He W, Smith A, et al. (2007) CCL3L1 and CCR5 influence cell-mediated immunity and affect HIV-AIDS pathogenesis via viral entry-independent mechanisms. Nat Immunol 8: 1324-1336.

5. Gonzalez E, Bamshad M, Sato N, Mummidi S, Dhanda R, et al. (1999) Race-specific HIV-1 disease-modifying effects associated with CCR5 haplotypes. Proc Natl Acad Sci U S A 96: 12004-12009.

6. Ahuja SK, Kulkarni H, Catano G, Agan BK, Camargo JF, et al. (2008) CCL3L1-CCR5 genotype influences durability of immune recovery during antiretroviral therapy of HIV-1-infected individuals. Nat Med 14: 413-420.

7. Quinn TC, Wawer MJ, Sewankambo N, Serwadda D, Li C, et al. (2000) Viral load and heterosexual transmission of human immunodeficiency virus type 1. Rakai Project Study Group. N Engl J Med 342: 921-929.

8. Gray RH, Wawer MJ, Brookmeyer R, Sewankambo NK, Serwadda D, et al. (2001) Probability of HIV-1 transmission per coital act in monogamous, heterosexual, HIV-1-discordant couples in Rakai, Uganda. Lancet 357: 1149-1153.

9. Operskalski EA, Stram DO, Busch MP, Huang W, Harris M, et al. (1997) Role of viral load in heterosexual transmission of human immunodeficiency virus type 1 by blood transfusion recipients. Transfusion Safety Study Group. Am J Epidemiol 146: 655-661.

10. Pedraza MA, del Romero J, Roldan F, Garcia S, Ayerbe MC, et al. (1999) Heterosexual transmission of HIV-1 is associated with high plasma viral load levels and a positive viral isolation in the infected partner. J Acquir Immune Defic Syndr 21: 120-125.

11. Fideli US, Allen SA, Musonda R, Trask S, Hahn BH, et al. (2001) Virologic and immunologic determinants of heterosexual transmission of human immunodeficiency virus type 1 in Africa. AIDS Res Hum Retroviruses 17: 901-910.

12. Wawer MJ, Gray RH, Sewankambo NK, Serwadda D, Li X, et al. (2005) Rates of HIV-1 Transmission per Coital Act, by Stage of HIV-1 Infection, in Rakai, Uganda. J Infect Dis 191: 1403-1409.

13. Hickie C, Hickie I, Silove D, Wakefield D, Lloyd A (1995) Delayed-type hypersensitivity skin testing: normal values in the Australian population. Int J Immunopharmacol 17: 629-634.

14. Hanley JA (2001) A heuristic approach to the formulas for population attributable fraction. J Epidemiol Community Health 55: 508-514.

15. Rivas AL, Tennenbaum SE, Aparicio JP, Hoogesteijn AL, Mohammed HO, et al. (2003) Critical response time (time available to implement effective measures for epidemic control): model building and evaluation. Can J Vet Res 67: 307-311.

16. Ball F, Neal P (2002) A general model for stochastic SIR epidemics with two levels of mixing. Math Biosci 180: 73-102.

17. van Nes A (2001) Mathematical modelling of pseudorabies virus (syn. Aujeszky's disease virus) outbreaks aids eradication programmes: a review. Vet Q 23: 21-26.

18. Wang D, Zhao X (2003) [Empirical analysis and forecasting for SARS epidemic situation]. Beijing Da Xue Xue Bao 35 Suppl: 72-74.

19. De Jong MCM, Diekmann O, Heesterbreek JAP (1993) The contribution of Ro for discrete-time epidemic models with dynamic heterogeneity. Centrum voor Wiskunde en Informatica.

20. Hyman JM, Li J (2000) An intuitive formulation for the reproductive number for the spread of diseases in heterogeneous populations. Math Biosci 167: 65-86.

21. Hill AN, Longini IM, Jr. (2003) The critical vaccination fraction for heterogeneous epidemic models. Math Biosci 181: 85-106.
