# Supplementary material for: Role of CCL3L1-CCR5 Genotypes in the Epidemic Spread of HIV-1 and Evaluation of Vaccine Efficacy
Source: PLoS One. 2008 Nov 7;3(11):e3671. doi: 10.1371/journal.pone.0003671 (PMC2576446; doi:10.1371/journal.pone.0003671)
Supplement: Table S1 — Summary of parameters used to model the influence of CCL3L1-CCR5 GRGs on epidemiological endpoints. Note, the inclusion of the parameters of vaccine efficacy and durability were included on a proof-of-principle basis for vaccines which rely in part on CMI for their effects. (0.03 MB DOC) [file pone.0003671.s002.doc]

**Table S1. Summary of parameters used to model the influence of *CCL3L1-CCR5* GRGs on epidemiological endpoints.** Note, the inclusion of the parameters of vaccine efficacy and durability were included on a proof-of-principle basis for vaccines which rely in part on CMI for their effects.

| ***Parameter*** | ***Description*** | ***Range of Values assumed (*) or obtained from data*** |
| --- | --- | --- |
| ß_u_ | Annual transmission probability based on the influence of the GRGs on VL set points, and is a measure of the degree of infectiousness associated with each GRG. | 0.0655 – 0.1620 |
| ß_a_ | Annual transmission probability that factors in the following: (i) ß_u_; (ii) the duration of infectiousness and for this we used the adjusted RHs for progression to AIDS for GRGs of the infected partner; and (iii) the risk of HIV acquisition based on the GRGs of the susceptible partner. | 0.0655 – 0.3866 |
| *R_o_* | Basic reproductive number estimated from ßa and accounting for an assumed background death rate of 0.025 and an estimated incidence 0.043 for AIDS. | 0.96 – 5.69 |
| *e* | Vaccine efficacy is the product of the *take* and *degree*. We only accounted for the influence of the GRGs on the take (t). These value ranges are empiric and were used for the sensitivity analyses. | 0.3 – 0.7^*^ |
| *t* | Relative vaccine take across the GRGs. Estimated from the mean best DTH responses across GRGs and normalizing these values to the mean DTH response of subjects belonging to the low risk GRG. | 1, 0.94 and 0.86 for low, moderate and high risk GRGs |
| *f* | Fraction of subjects in whom the vaccine efficacy does not wane i.e. vaccine durability. Estimated by assuming that the duration of vaccine protection in the low risk GRG is 10 years and by estimating the odds of anergy (failure to respond to vaccine) in the other GRGs. The values of *f* in the adjacent column to the right indicate that the duration of vaccine protection is 10, 5.6 and 3.9 years in the low, moderate and high risk GRGs, respectively. (Section 1.4) | 0.9, 0.82 and 0.74 for low, moderate and high risk GRGs. |
| *Pc* | Critical proportion of the population- or cohort-based vaccination coverage required to limit the epidemic. | 0 – 2.59 |
