# Supplementary material for: Role of CCL3L1-CCR5 Genotypes in the Epidemic Spread of HIV-1 and Evaluation of Vaccine Efficacy
Source: PLoS One. 2008 Nov 7;3(11):e3671. doi: 10.1371/journal.pone.0003671 (PMC2576446; doi:10.1371/journal.pone.0003671)

**Table S2. Results of mathematical modeling of the influence of *CCL3L1-CCR5* GRGs on epidemiological endpoints.** Group, indicates subdivision of the population into 9 groups based on their GRGs (Figure 2a). Estimated frequency indicates the proportion of the population groups from data derived from the WHMC cohort. The parameters are described in Supplementary Table S1.


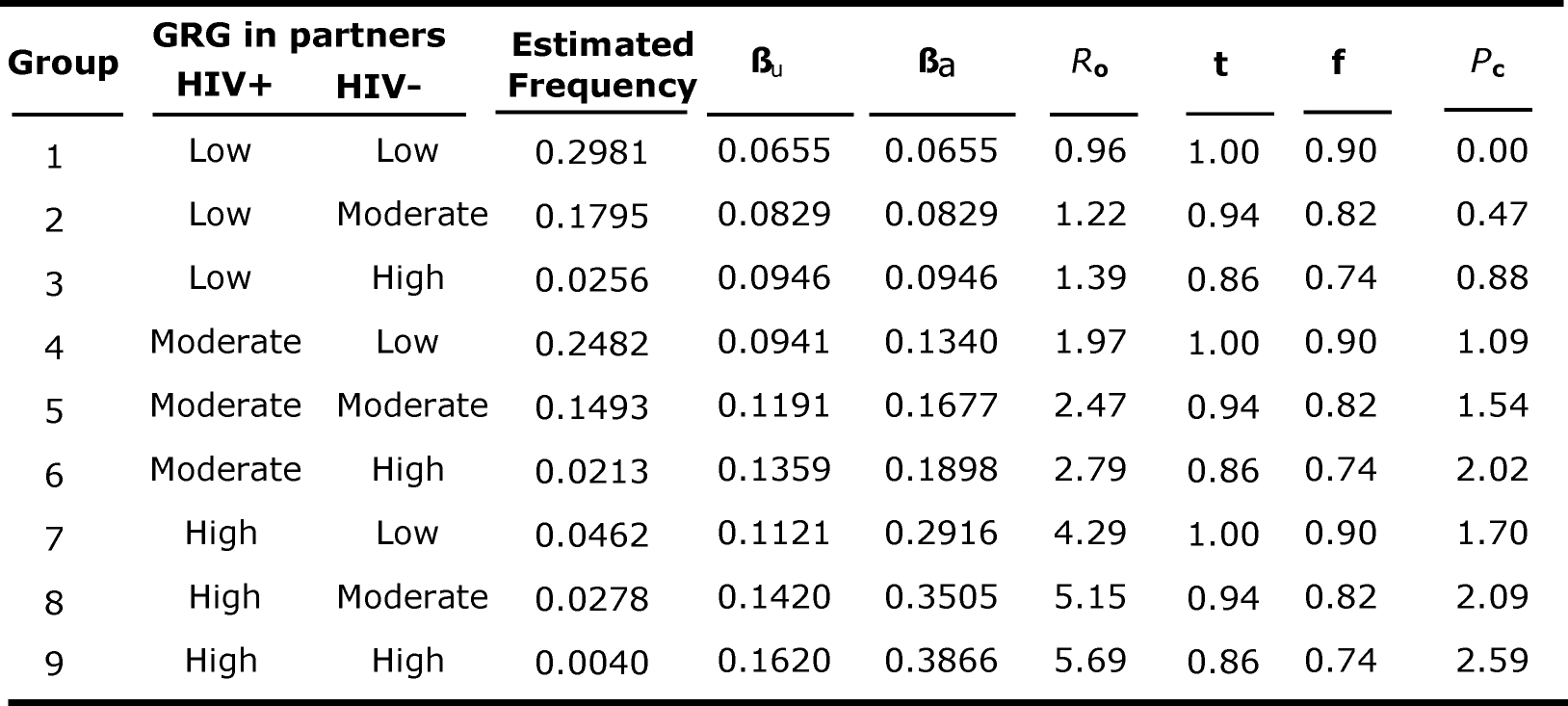

Supplement: Table S2 — Group, indicates subdivision of the population into 9 groups based on their GRGs (Figure 2a). Estimated frequency indicates the proportion of the population groups from data derived from the WHMC cohort. The parameters are described in Supplementary Table S1. (0.05 MB DOC) [file pone.0003671.s003.doc]
